# Anti-Viral Potential and Modulation of Nrf2 by Curcumin: Pharmacological Implications

**DOI:** 10.3390/antiox9121228

**Published:** 2020-12-04

**Authors:** Mahdie Rahban, Mehran Habibi-Rezaei, Mansoureh Mazaheri, Luciano Saso, Ali A. Moosavi-Movahedi

**Affiliations:** 1 Institute of Biochemistry and Biophysics, University of Tehran, Tehran 1417614335, Iran; mrohban@ut.ac.ir; 2 School of Biology, College of Science, University of Tehran, Tehran 1417614335, Iran; 3 Center of Excellence in NanoBiomedicine, University of Tehran, Tehran 1417614335, Iran; 4 Research Center of Food Technology and Agricultural Products, Department of Food Toxicology, Standard Research Institute, Karaj 3158777871, Iran; m_mazaheri@standard.ac.ir; 5 Department of Physiology and Pharmacology “Vittorio Erspamer”, Sapienza University of Rome, P. le Aldo Moro 5, 00185 Rome, Italy; luciano.saso@uniroma1.it; 6 UNESCO Chair on Interdisciplinary Research in Diabetes, University of Tehran, Tehran 1417614335, Iran

**Keywords:** Nrf2, curcumin, Keap1, viral infection, acute and chronic inflammation diseases, bioavailability, oxidative stress, COVID-19, pharmacological applications

## Abstract

Nuclear factor erythroid 2-related factor 2 (Nrf2) is an essential transcription factor that maintains the cell’s redox balance state and reduces inflammation in different adverse stresses. Under the oxidative stress, Nrf2 is separated from Kelch-like ECH-associated protein 1 (Keap1), which is a key sensor of oxidative stress, translocated to the nucleus, interacts with the antioxidant response element (ARE) in the target gene, and then activates the transcriptional pathway to ameliorate the cellular redox condition. Curcumin is a yellow polyphenolic curcuminoid from *Curcuma longa* (turmeric) that has revealed a broad spectrum of bioactivities, including antioxidant, anti-inflammatory, anti-tumor, and anti-viral activities. Curcumin significantly increases the nuclear expression levels and promotes the biological effects of Nrf2 via the interaction with Cys151 in Keap1, which makes it a marvelous therapeutic candidate against a broad range of oxidative stress-related diseases, including type 2 diabetes (T2D), neurodegenerative diseases (NDs), cardiovascular diseases (CVDs), cancers, viral infections, and more recently SARS-CoV-2. Currently, the multifactorial property of the diseases and lack of adequate medical treatment, especially in viral diseases, result in developing new strategies to finding potential drugs. Curcumin potentially opens up new views as possible Nrf2 activator. However, its low bioavailability that is due to low solubility and low stability in the physiological conditions is a significant challenge in the field of its efficient and effective utilization in medicinal purposes. In this review, we summarized recent studies on the potential effect of curcumin to activate Nrf2 as the design of potential drugs for a viral infection like SARS-Cov2 and acute and chronic inflammation diseases in order to improve the cells’ protection.

## 1. Introduction

Under healthy conditions, there is a balance between antioxidant defense systems and free radical generation. However, under impaired balance between antioxidants and oxidants, oxidative stress develops, and cellular damage occurs [1], which is featured by multiple pathological chronic diseases, and the severity of viral infections like as recently pandemic of coronavirus disease 2019 (COVID-19) as a contagious respiratory and vascular disease [2]. Nrf2 (nuclear factor erythroid-derived 2-like 2 (*NFE2L2*)), as a stress-responsive transcription factor, effectively activates gene products and reduces reactive oxygen species (ROS) and electrophiles; thereby, the progression of various types of chronic diseases are prevented or retarded [3,4]. Keap1 rigorously regulated Nrf2, a key sensor of oxidative stress, and Cullin 3, in order to keep Nrf2 in the cytoplasm and actively present it for ubiquitination and proteasomal degradation [5]. Under oxidative stress, Keap1 releases Nrf2, which leads its translocation from cytosol to cellular nucleus, resulting in the expression of the antioxidant enzymes through an association with the antioxidant response element (ARE) and small musculoaponeurotic fibrosarcoma (sMaf) in order to combat oxidative stress and maintain cellular redox homeostasis [6,7]. Thereby, Nrf2 and associated proteins account as an ideal target against oxidative stress-related diseases, including neurodegenerative diseases, cancers, viral infection, cardiomyopathy, insulin resistance, and ischemia-reperfusion injury, as well as to protect other tissues or organs by regulating its level [8,9]. Nowadays, nutraceuticals and functional foods, including natural compounds, have been highlighted more than ever toward health keeping, disease prevention, and treatment [10]. Among them, curcumin (also called diferuloylmethane) is the main natural polyphenol of turmeric that has been traditionally used, due to its anti-inflammatory, anti-diabetic, anti-oxidant, anti-microbial, and anti-carcinogenic properties [11,12,13,14,15,16]. It has been shown that curcumin mediates the mitochondrial dysfunction inhibition and Nrf2 release/translocate into the nucleus in order to regulate the cell antioxidant pathways and provide cell survivability [9,17]. Here, we summarize the recent studies on the Nrf2 activation by curcumin and its derivatives as potential drugs and then provide a brief overview of strategies in order to improve curcumin bioavailability in the target cells with the aim of its clinical applications. 

## 2. Oxidative Stress-Related Pathogenesis

Oxidative stress is a condition of unbalance between the generation and clearance ROSs in cells, which occurs under the disability of the cellular antioxidant system to detoxify them. The ROSs comprises radical species, including superoxide (O_2_^•−^), peroxide (O_2_^•2−^), hydroxyl (HO^•^), peroxyl (RO_2_^•^), hydroperoxyl (HO_2_^•^), alkoxyl (RO^•^), and non-radical species, including hydrogen peroxide (H_2_O_2_), hydroxyl ion (OH^−^), ozone (O_3_), oxygen singlet (^1^O_2_), and organic hydroperoxide (ROOH) [17,18]. They are generated either normally or because of the presence of toxicants or pathologic insults [19,20]. Although ROSs generation occurs in the various cell compartments, mitochondria are the prime. Nevertheless, ROSs are potentially produced all over either cell in molecular oxygen, intentionally or non-intentionally utilizing cellular processes [21,22,23]. At oxidative stress conditions, intracellular ROSs promote cell inflammation and pathogenesis of a broad range of oxidative diseases and, more importantly, insulin resistance disease, including metabolic and cognitive disorders [24,25]. ROS pathology is executed through the activation of redox-sensitive transcription factors, such as nuclear factor-*κ*B and activator protein 1 (NF-*κ*B and AP-1, respectively), in order to upregulate kinases, including mitogen-activated protein kinases, extracellular signal-regulated kinase, c-JUN N-terminal kinase phosphoinositide 3-kinase (MAPKs, ERK, and JNK, PI3K, respectively), and p38. The improper activation of NF-*κ*B also relates to other inflammatory diseases, such as atherosclerosis, inflammatory bowel disease, rheumatoid arthritis, asthma, and Helicobacter pylori-associated gastritis [4].

## 3. The Keap1–Nrf2 Pathway

The Keap1–Nrf2 pathway plays a crucial role in protecting cells against endogenous and exogenous oxidative stresses and xenobiotic damages [26,27]. Kelch ECH associating protein 1 (Keap1) is the key component of the pathway. However, some accessory proteins also contribute to the cytoprotective function of the process, including ARE and sMaf proteins [28].

The Nrf2 is a transcription factor and it belongs to cap ‘n’ collar (CNC) basic leucine zipper (bZIP) protein subfamilies. The human Nrf2 comprises 605 amino acid residues, which is folded in seven highly conserved Nrf2-ECH (Neh1-7) domains [29,30]. Among them, the Neh1 domain contains a bZIP motif, which mediates dimerization and DNA binding while using leucine zipper (L-Zip) and a basic region structure, respectively. Neh2 plays a role in rapidly binding to Keap1 that is mediated by ETGE and DLG motifs (representing high and low binding affinities, respectively) and Keap1-dependent ubiquitination and degradation that are mediated by a region of seven lysine residues and the Keap1–Cul3 E3 ligase complex, and then this complex degrade by the 26S proteasome. Cullin 3 (Cul3) is a subunit of the E3 ligase complex that has a role as a scaffolding protein to keep close Rbx1 (RING-box protein 1)-Ub (ubiquitin)-loaded E2 and Keap1. Additionally, the 26S proteasome degrades Nrf2 by recognizing the phosphorylation of specific serine residues in the Neh6 domain of Nrf2. This process keeps the cellular Nrf2 at low levels [31,32]. Nrf2 has a rapid turnover and it presents a half-life of about 20–30 min. due to its constant degradation by the ubiquitin-proteasome system [33]. The Neh6 domain plays the role of a degron to mediate Nrf2 degradation in the nucleus mediating by phosphorylation of sites at specific serine residues [34]. Keap1–Nrf2 binding has been modeled as “hinge and latch”. According this model, the Kelch domain of the Keap1 at β- propeller conformation binds to the Neh2 domain of Nrf2 protein through high- and low-affinity bindings to the ETGE motif (as hinge) and DLG motifs (as latch), respectively [33].

Keap1 is a member of the BTB (bric-a-brac, tram-track, and broad-complex) Kelch family of proteins, which consists of 624 amino acid residues. Keap1, which is a zinc-metalloprotein with 27 cysteine residues, has five structural regions: N-terminal region (NTR), BTB domain, intervening region (IVR), Kelch domain, and C-terminal region (CTR). The BTB domain mediates the homodimerization of Keap1 and Cullin3 (Cul3) binding contains a sensor Cys151 residue. Additionally, IVR also contains a number of reactive cysteine residues, including Cys273 and Cys288. The Kelch domain with a six-bladed β -propeller conformation is responsible for Keap1 binding to the DLG and ETGE motifs in the Neh2 domain of Nrf2. Keap1 binds to Cul3 through both its BTB and IVR domains, targeting substrates for ubiquitination [31,35,36,37]. Cysteine residues, including Cys151, Cys273, and Cys288, are targets of electrophiles and oxidants, and their modification pattern leads to conformational changes in the Keap1, which disrupts the interaction between the Nrf2 that is mediated by Neh2 domain and Keap1 Kelch domains in order to remove the suppression of Nrf2 and then cancel its polyubiquitination [38]. Cys273 and Cys288 control basal and stress conditions of the cells, whereas, under cellular stress, Cys151 locates in the BTB domain is further reactive; likewise, specific toxins appear to modify Cys226, Cys434, and Cys613, thus disrupting the Keap1–Nrf2 complex [38,39]. The two cysteine residues, Cys273 and Cys288, in the central linker domain of Keap1, are involved in repressing Nrf2-dependent transcriptional activation [40]. Thereby, cysteine modification could be used as a therapeutic strategy for modulating the Keap1–Nrf2 complex. Natural compounds, such as curcumin, could be a potential drug to control the cell signaling pathway in stress conditions through Cys151 modification [31].

Under the normoxidant status of the cell, Keap1 keeps and suppresses Nrf2 in the cytosol in order to facilitate its ubiquitination by its E3 ligase activity in assembly with Cul3 and degradation in 26S proteasome. Meanwhile, under oxidative stress or electrophilic status, the modification of Keap1 at three candidate cysteine residues brings about altering the conformation of Keap1, which weakens Keap1–DLG, and, as a result, Nrf2 detachment and translocation to the nucleus [31]. In the nucleus, Nrf2 combines with the sMaf protein in order to form a heterodimer to activate ARE, which results in increasing the expression of a list of antioxidant enzymes [8]. The list includes heme oxygenase-1 (HO-1), NADP(H) quinone oxidoreductase 1 (NQO1), catalytic subunit (GCLC), glutamate-cysteine ligase modifier subunit (GCLM), CAT, SOD, thioredoxin reductase 1 (TrxR1), and aldo-keto reductase1 subunits C-1 (AKR1C1), C-2 (AKR1C2), and C-3 (AKR1C3) [3,8,41]. Moreover, plenty of functions are activated, including redox regulation, protein homeostasis, mitochondrial physiology, amino acid metabolism, iron metabolism, DNA repair, and some cellular processes, are prevented including apoptosis, autophagy, and proteasomal degradation [1,32].

## 4. Keap1/Nrf2 Targeted Drug Design

The Keap1–Nrf2-ARE pathway shows a vital role in protecting cells from oxidative stress, which results in numerous human diseases. Targeting Nrf2 activation in the context of protein–protein interactions (PPIs) has emerged as an attractive strategy for designing innovative drugs to modulate Keap1–Nrf2 signaling and the transcription and expression of cytoprotective proteins, which is crucial in preventing oxidative stress-related pathological conditions. The Keap1–Nrf2 interaction inhibitors have been shown to be beneficial in many clinical indications. Direct disruptor or modifiers of the Keap1–Nrf2 complex could interrupt or induce conformational changes in the Keap1 structure. Later, Nrf2 is released and then moved from cytosol into the nucleus in order to activate the downstream antioxidant response element pathway. Accordingly, drug design-based Nrf2 activation strategies can be divided into modifying Keap1, leading to its conformational changes and disturbance of the Keap1–Nrf2. Numerous research studies described various Keap1–Nrf2 inhibitors, Keap1 modifiers, and Nrf2 activators [8,42].

Small molecules, like analogs of naphthalene-based or N-biphenyl substituted pyrazole carboxylate, have the potency to inhibit Keap1–Nrf2 interactions at the nanomolar scale, subsequently providing drug-like properties [43]. Dimethyl fumarate (DMF, Tecfidera), which is clinically used for the treatment of MS (Multiple sclerosis), is one of the modifiers of the Keap1–Nrf2 complex and it could covalently bind to the Cys151 residue of the Keap1–BTB domain. Bardoxolone (CDDO) reversibly reacts with the Cys151 residue of Keap-1, is designed to treat kidney disease in diabetic patients, but failed in phase 3 clinical trial due to the associated cardiac disease risks [8,35]. Although some of these small molecules are able to bind Keap1 at nanomolar potency, they exhibit moderate or even no inhibition activity against the Keap1–Nrf2 interaction. Hence, not every binding potency of Keap1 indicates Keap1–Nrf2 inhibition. There are several ETGE-like motifs in the Keap1 structure, and most of the inhibitors that bind to them cannot inhibit the interactions between Keap1–Nrf2 [44]. 

The proteasomal degradation of Nrf2 could be disrupted by some electrophiles that bind to Keap-1 sulfhydryl groups of the cysteine residues. Sulforaphane and tert-butyl hydroquinone (electrophilic substances) could react with the Cys151 and Cys171 of Keap1 and then change the ligase conformation in order to promote the escape of Nrf2 from degradation [45]. 

Peptide-based blockers have been designed to disrupt the Keap1–Nrf2 interactions, but low membrane permeability, poor bioavailability, instability, and high polarity are the major limitations of their application as a drug. [46,47]. Among designed peptide blockers, two egg-derived peptides, DKK and DDW, and the indole ring of tryptophan tetrapeptide W4 like cyclic motif-based peptide have been reported to disrupt Keap1–Nrf2 interaction, but additional validation is needed [44]. Fragment-based Kelch domain peptides that could interrupt the direct interaction of Keap1–Nrf2 is designed. These peptides bind to Keap1 with nanomolar affinity [48].

**Figure 1:**
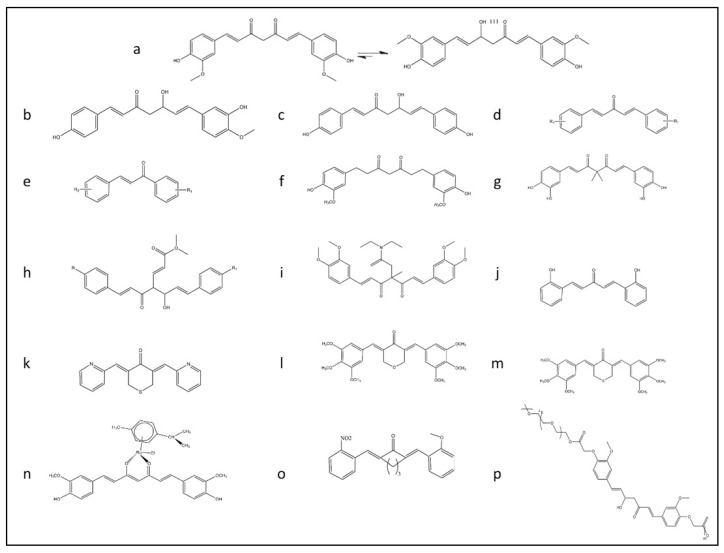
The rational design of derivative of curcumin for improving the bioavailability of this structure and promoting Nrf2 signaling pathway summarized in this figure. (**a**) Keto-enol form of curcumin structure. (**b**) Deoxymethylcurcumin. (**c**) Bisdeoxymethylcurcumin. (**d**) 5-carbon enone spacer analog of curcumin. (**e**) 3-carbon enone spacer analog of curcumin. (**f**) Tetrahydrocurcumin analog. (**g**) Curcumin analog with the geminal dimethyl groups and the catechol moiety. (**h**) Fumarate and curcumin-based analog. (**i**) TML-6 analog with modification for fix isomerization of keto form of curcumin with a methyl group and N, N-diethylacetamide. (**j**) Modified form of curcumin analog with five carbon linkages bis[2-hydroxybenzylidene] acetone (BHBA). (**k**) FN1 a synthetic analog of curcumin ((3E,5E)-3,5-bis(pyridin-2/3/4-methylene)-tetrahydrothiopyran-4-one). (**l**) E10 a synthetic analog of curcumin ((3E,5E)-3,5-bis-(3,4,5-trimethoxy-benzylidene)-tetrahydropyran-4-one). (**m**) F10 a synthetic analog of curcumin ((3E,5E)-3,5-bis-(3,4,5-trimethoxybenzylidene)-tetrahydro-thiopyran-4-one). (**n**) Ruthenium (II)-curcumin compound. (**o**) A13 a synthetic analog of curcumin (2,6-bis((3-methoxy-4-hydroxyphenyl) methylene)-cyclohexanone). (**p**) PEGylated analog of curcumin.

**Figure 2:**
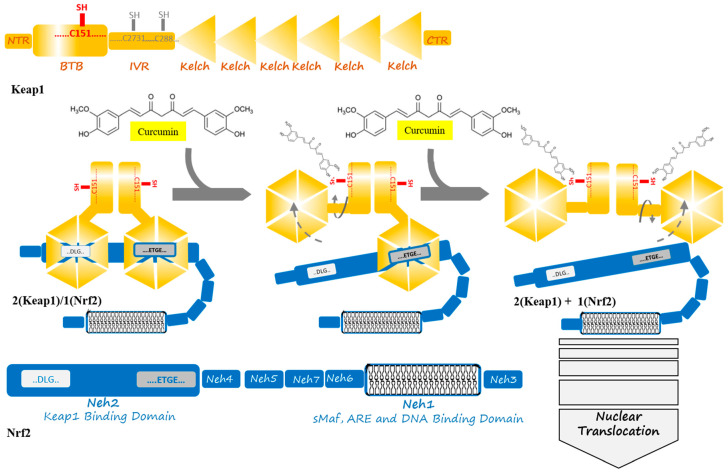
Curcumin induces a change in Keap1 conformation through binding to the sensor C151 residue of the Keap1 at the BTB domain. This binding is supposed to result in successive breaking of low- and high-affinity interactions between the Kelch domain of Keap1 and DLG- and ETGE motifs from Nrf2. The released Nrf2 is able to translocate to the cell nucleus to act as a transcription factor where starts anti-oxidant enzymes expression in an ARE and sMaf collaboration.

Natural products, like curcumin, contain enone and ketone groups as Michael acceptors, which makes it prone to react with the key cysteine thiolate residues in Keap1 (Figure 1a) [49]. Figure 1 shows the structure of the curcumin and some of its derivatives and formulations. According to the mass spectroscopy analysis-data curcumin modifies Cys151 of Keap1, resulting in conformation change to negatively impact the Keap1–Nrf2 PPI (Figure 2). Curcumin treatments of the cells with the mutations in Keap1–C273S, Keap1–C288S, and Keap-1-Cys151S could not activate Nrf2 translocation. Consequently, curcumin as an Nrf2 activator could be a potential drug to protect cells and although it has been considered as a natural therapeutic agent in various traditional medicines, but further clinical investigation is needed.

## 5. Nrf2 Targeting by Curcumin as a Therapeutic Agent

People around the world, for different remedies purposes, have consumed natural products. Turmeric is one of the most popular medicinal herbs with a long history of administration in India, Iran, and China. It has traditionally been applied to treat a broad range of diseases [50,51]. It is also commonly used in some Asian and African countries as a dietary spice. Curcumin is a hydrophobic polyphenol yellow pigment obtained from the dried rhizomes, of *Curcuma longa*, a rhizomatous herbaceous plant that belongs to the ginger family (*Zingiberaceae*) and it is a primary active compound of turmeric [11,12]. Curcuminoids complex, which is found in turmeric, consists of curcumin, demethoxycurcumin, and bisdemethoxycurcumin (Figure 1a–c, respectively) [13].

Extensive research shows that curcumin has a wide admiration range of beneficial properties, including anti-inflammatory, anti-diabetic, anti-oxidant, anti-microbial, anti-arthritic, anti-carcinogenic activity, and wound healing effects [14,15,16]. In vitro and in vivo studies demonstrated the ability of curcumin to modulate multiple cellular targets. Curcumin could modify multiple cell signaling pathways and downregulates the cell survival gene expression profile by its effects on transcription factors [52]. Curcumin affects the PI3K/Akt-1/mTOR, the Ras/Raf/MEK/ERK, the GSK-3β pathway, activates the p53 pathway, and regulates survival pathways via NF-κB, Akt, sand Nrf2/ARE pathways [53,54]. Curcumin exhibits antioxidant activity, because of donating hydrogen and scavenging free radicals. Besides, curcumin inhibits lipo-oxygenase (LOX) and cyclo-oxygenase (COX), xanthine oxygenase activities, nitric oxide synthesis, and ROS generation. Curcumin also inhibits the production of pro-inflammatory monocyte/macrophage-derived cytokines [interleukin- 8 (IL-8), monocyte inflammatory protein-1 (MIP-1), monocyte chemotactic protein-1 (MCP-1), interleukin-1b (IL-1b), and tumor necrosis factor-α (TNF-α) [55,56]. Consequently, curcumin is a non-toxic natural product, and its broad range of beneficial activities could be utilized as a therapeutic agent in various diseases.

Curcumin is able to suppress acute and chronic inflammation [11,55] and it has been asserted to penetrate the blood-brain barrier and moderate cerebral edema, reduce the inflammatory response, influence synaptic plasticity, and improve energy homeostasis through its direct and indirect antioxidant effects by eliminating ROS. Additionally, curcumin induces the expression of cytoprotective proteins through the Keap1–Nrf2 signaling pathway [57]. Besides, curcumin protects normal organs and sensitizes cancer cells through the activation of Nrf2. Nrf2 under activation by curcumin could inactivate the NF-*κ*B and AP-1 signaling pathways, resulting in competitively blocking their activation by various stimuli [4,58]. Many recent studies have reported that oxidative stress, inflammation, and matrix degradation are essential contributors in arthritis, including temporomandibular joint (TMJ) osteoarthritis as a common stomatognathic disease. The curcumin treatment of human TMJ osteoarthritis effectively activates the Nrf2/ARE pathway in a dose-dependent manner [59]. The administration of curcumin significantly decreases H_2_O_2_ that is induced by oxidative damages and cell toxicity in osteoblast-like cells via retaining the glycogen synthase kinase 3 beta (GSK3β)-Nrf2 signaling pathway, which provides a possible promising osteoporosis treatment strategy [60]. The curcumin treatment of rat pheochromocytoma-derived cell line (PC12) exhibits a reduction of H_2_O_2_ that is induced by oxidative damages of DNA and increases cell viability under exposure to H_2_O_2_ [61]. Moreover, related studies to the current SARS-CoV-2 outbreak show that oxidative stress is associated with viral infection and pathogenesis. Therefore, the activation of antioxidant response genes and increased expression of heme oxygenase-1 (HO-1) and NAD(P)H:quinone oxidoreductase (NQO-1) through the Nrf2/ARE signaling pathway makes it a possible therapy strategy [62,63].

## 6. Formulation and Functional Improvement of Curcumin 

Naturally occurring constituents of turmeric contain 3–6% curcuminoids, which is responsible for the yellowish color. Although more than 50 curcuminoids have been identified in turmeric rhizome, the main characteristic components make a ternary complex of curcumin (diferuloylmethane; 1,7-bis(4-hydroxy-3-methoxyphenyl)-1,6-heptadiene-3,5-dione), deoxymethylcurcumin, and bisdeoxymethylcurcumin at 94%, 6%, and 0.3%, respectively (Figure 1a–c) [13]. Curcumin contains two functional phenolic groups, and seven-carbon dienone spacer with a linear diarylheptanoid structure [64].

The main obstacles that are correlated with curcumin utilization are low bioavailability due to the chemical instability, insignificant solubility in water, low absorption, and rapid metabolism and elimination. Bioaccessibility is a crucial issue for curcumin to exert biological function. Curcumin must be absorbed by epithelium cells of the gastrointestinal tract before being transported into systemic circulation. Due to curcumin’s hydrophobic property, the small intestine epithelium cells could not absorb the curcumin in oral consumption. Moreover, due to the rapid metabolism curcumin degradation occurs in the liver and small intestine before reaching the systemic circulation [65,66,67,68]. Hence, this pharmacokinetic limitation restrains the use of curcumin in clinics. Curcumin derivatives with different chemical groups have been synthesized in order to improve its solubility suitable for drug formulation. Moreover, different pharmaceutical and formulation strategies for oral administration of curcumin involving polymeric micelles, solid dispersions, nanosuspensions, cyclodextrins, lipid-based nanocarriers, polymorphs, nano/microparticles, and curcumin conjugates have been developed to increase the bioavailability of curcumin pharmacological application in recent years [14,69]. 

All enone analogs of curcumin, including 5-carbon enone spacer and 3-carbon enone spacer (Figure 1d,e), could modify Keap1 through Michael addition and activate Nrf2 [70], but it has been reported that tetrahydrocurcumin (THC) (Figure 1f), a non-electrophilic analog of curcumin, could not induce nuclear translocation of Nrf2 [71]. 

Tu’s group designed and synthesized curcumin analog with the geminal dimethyl groups and the catechol moieties (Figure 1g). This analog has improved curcumin stability and increased the cell’s cytoprotective effect by induction of Nrf2 activation in a Keap1-dependent manner [72]. 

A recent study has proposed that the level of α-Synuclein and phosphorylated tau protein (p-tau proteins) decreased by the activation of Nrf2. The overexpression of the GSK-3β and downregulation of the Nrf2/ARE pathway attenuates the cytoprotective effects of cells and promotes the development of neurodegenerative disorders. Therefore, a dual modulator of GSK-3β inhibitor/Nrf2 inducers of fumarate and curcumin-based analog (Figure 1h) has been designed, and it has exhibited significant neuroprotective effects to PD therapeutics [73]. 

TML-6 (a modified form of traditional curcumin) has improved curcumin’s stability and metabolism, but it has low water solubility like curcumin. SinoPharm Pharmaceutical Company (SPT) has synthesized TML-6 ((1E,6E)-1,7-bis(3,4-dimethoxyphenyl)-4,4-disubstituted-hepta-1,6-diene-3,5-dione), wherein the four position was substituted with a methyl group and N, N-diethylacetamide (Figure 1i). The slowdown in metabolism has been derived by replacing methoxyl groups on curcumin’s benzene structure. Furthermore, TML-6 was more stable in acidic pH than curcumin due to of fixed isomerization. Cell biological studies have demonstrated that TML-6 could activate the Nrf2 gene in a dose-dependent manner, being 12.1 fold stronger than the traditional curcumin. TML-6 has suppressed NF-*κ*B and mTOR, upregulated Apo E, and inhibited the synthesis of the β-amyloid precursor protein and β-amyloid (Aβ). In animal models, treatment by TML-6 significantly improved learning, reduced Aβ, and suppressed microglial activation marker Iba-1 in the brain [74]. 

In one study, a curcumin analog with five carbon linkages bis[2-hydroxybenzylidene] acetone (BHBA) (Figure 1j) has been synthesized in order to induce Nrf2. BHBA has interacted with Cys151 of Keap1 and activated the Nrf2 pathway with minimal toxicity and noticeably reduced lung adenocarcinoma; thus, it could develop as a chemoprotective pharmacological agent [75]. 

The treatment of prostate cancer cells with FN1 ((3E,5E)-3,5-bis(pyridin-2/3/4-methylene)-tetrahydrothiopyran-4-one), a synthetic analog of curcumin (Figure 1k), has increased the level of Nrf2, decreased the level of Keap1, and activated the Nrf2/ARE signaling pathway. Hence, FN1 could be a cancer chemopreventive agent for the remediation of prostate cancer initiation, progression, and development [76]. 

Two synthetic curcumin derivatives—E10 ((3E,5E)-3,5-bis-(3,4,5-trimethoxy-benzylidene)-tetrahydropyran-4-one) (Figure 1l) and F10 ((3E,5E)-3,5-bis-(3,4,5-trimethoxybenzylidene)-tetrahydro-thiopyran-4-one) (Figure 1m)—have been synthesized by Li’s group. These derivatives have promoted the mRNA and protein level of Nrf2 and they have activated the Nrf2/ARE pathway in a transgenic adenocarcinoma mouse prostate (TRAMP) model of prostate cancer. F10 could reactivate the Nrf2 by hypomethylated the Nrf2 promoter [77]. 

Ruthenium (II)-curcumin compound (Figure 1n), as designed by Garufi’s group, induced a certain degree of cell death in all tested cancer cell lines, as well as Nrf2 activation, independent of the p53 status [78]. 

Xiang et al. investigated the biological properties of a newly synthesized curcumin analog A13 (2,6-bis((3-methoxy-4-hydroxyphenyl) methylene)-cyclohexanone) in the animal model of diabetic cardiomyopathy (Figure 1o). The histological lesions of the myocardium in diabetic rats have decreased, fibrosis in myocardium has ameliorated, superoxide dismutase activity has increased, and the level of malondialdehyde has decreased by treatment with curcumin and A13. Molecular analysis has demonstrated that a dose of 20 mg/kg of A13 could activate the Nrf2/ARE pathway and promote the nuclear translocation of Nrf2 with a better performance than curcumin [79].

The polymerized form of Nano-Curcumin (PNC) was used in order to treat autoimmune encephalomyelitis (EAE) as an experimental model of multiple sclerosis (MS) in female Lewis rats. The results have shown that the expression of Nrf2 and HO-1 genes increased [80]. Pandy’s group synthesized the potential Nrf2 activator of PEGylated curcumin analog (Figure 1p). This copolymer could activate Nrf2 several folds higher than curcumin [81]. Curcumin encapsulated solid lipid nanoparticles have shown that Nrf2 activated and the mitochondrial complexes, cytochrome and glutathione levels, and superoxide dismutase activity increased in HD rats [82]. Besides, curcumin that is encapsulated in PLGA based-nanoparticulate formulation has shown that formulated curcumin could activate Nrf2 in the presence of H_2_O_2_. Hence, it might be proper to protect neurons against Alzheimer’s disease [83].

Nanocurcumin (curcumin nanoemulsion) and PGV-0 (curcumin analog) have reduced Dengue virus infection. Nanocurcumin improved cell uptake in comparison to curcumin [84,85]. Curcumin chitosan nanocomposite exhibited a better inhibition of entrance and replication of HCV in human hepatoma cells than curcumin [84]. The curcumin modified silver nanoparticles (cAgNPs) inhibited RSV infection in the viral cell attachment stage and directly inactivated the virus [86]. The encapsulation of curcumin in protein nanoparticles could impair cancer cells’ viability and increased oral bioavailability in rats. Lipocurc^®^ (liposomal, IV) nanoformulations of curcumin and Meriva^®^ curcumin-phosphatidylcholine phytosome complex of soy lecithin have improved the bioavailability of curcumin but, rapid systemic elimination of Lipocurc^®^, and several adverse effects of Meriva^®^ have limited their application as medicine [87]. The free curcumin that is encapsulated in a tri-lipid matrix (Capsule Longvida^®^) based on the Solid Lipid Curcumin Particle (SLCP) technology has enhanced water solubility, and the rapid metabolism of curcumin has improved its entry in the bloodstream and reaching the tissue target [88,89]. CurQfen^®^ is a combination of galactomannan soluble dietary fiber; hence, this formulation could enhance oral bioavailability and improve the prolonged-release and rapid metabolism in the gastrointestinal tract [90,91]. NovaSol^®^, which is a micellar formulation, CurcuWin^®^, a hydrophilic carrier of polyvinyl pyrrolidine, Biocurcumax^TM^, a formulation of curcumin and essential oils of turmeric, Theracurmin^TM^, a formulation of curcumin that is based a colloidal nanoparticle, and Curcumin C3 Complex^®^, which is a lipophilic cavity of cyclodextrins, are all curcumin formulations with the aim of improving the bioavailability properties of the curcumin [52,92].

Moreover, for effective curcumin delivery, genipin has stabilized a biocompatible complex of caseinate-chitosan nanoparticles. This encapsulated curcumin has been produced for improved cytotoxicity and cellular uptake in cancer cells [93]. Additionally, encapsulated curcumin in walnut protein has represented improving water solubility and antioxidant activity; further, it has been increased to release curcumin in the body’s gastrointestinal condition [94,95]. 

Many studies have been developed to design a liposome-encapsulated, milk-derived exosome complex of curcumin for effective delivery and they have improved cytotoxic action against cancer cells [96,97,98,99,100]. Curcumin loaded in whey protein aggregates (WPA) in the presence of *k*-carrageenan has exhibited a further protection of curcumin within the upper gastrointestinal tract and it could deliver to the colon. In addition, curcumin encapsulation with Gum Arabic (GA) and whey protein nanofibrils have increased the antioxidant activity and photostability of the curcumin besides a sustained-release profile in gastrointestinal conditions [101,102]. 

## 7. Amelioration of the Candidate Diseases through Nrf2 Modulation by Curcumin

Curcumin is a non-toxic compound to humans at high doses with numerous pharmacological activities and different cellular effects. Curcumin is a beneficial compound for introducing new drug design strategies in medicine when considering unique chemical structure and function for modulating multiple cell signaling pathways.

### 7.1. Kidney Diseases

The main element in the incidence of acute kidney injury and chronic kidney disease progression is an inflammation and oxidative damage of renal cells that promoted nephrotoxicity [103]. Nrf2, by eliminating ROS, improves kidney diseases as a considerable regulator of redox balance [3,104,105,106]. Nrf2 regulates cellular defense genes where is ROS elevated in the kidneys of wild-type mice, but this regulation is not observed in Nrf2- knock-out mice. Related experiments have indicated that curcumin could ameliorate inflammation and oxidative stress by activating the Nrf2/HO-1 signaling pathways [107]. Another study in renal epithelial cells has demonstrated that curcumin could stimulate the expression of Nrf2 in a time and dose dependent manner. Additionally, HO-1 protein expression and heme oxygenase activity were remarkably increased by the dissociation of the Nrf2-Keap1 complex [108]. Models of cisplatin-induced renal toxicity were used in order to investigate the effect of curcumin. The protective effect of curcumin could be mediated by the up-regulation of survival signals, like Akt and Nrf2/HO-1, and the moderation of KIM-1 and NF-*κ*B [103]. The stone formation in Nephrolithiasis produces by oxidative stress, inflammation, apoptosis, fibrosis, and autophagy. In the nephrolithiasis mouse model, oral administration of curcumin for 14 days could notably diminish glyoxylate and tissue injury in kidneys. Treatment with curcumin has significantly inhibited apoptosis and autophagy of mouse renal cells and it has also alleviated the high expression of IL-6, MCP-1, OPN, CD44, α-SMA, Collagen I, and collagen fibril deposition, which were promoted by hyperoxaluria. The oxidative stress response has reduced MDA content and increased SOD, CAT, GPx, GR, and GSH levels, by curcumin administration. Nrf2 has accumulated in the nucleus, and the production of HO-1, NQO1, and UGT have decreased in the kidneys of mice [109]. Curcumin treatment of the nephrectomized rat model has demonstrated that renal dysfunction is ameliorated by increasing expressions of Nrf2 and HO-1. In nephrectomized rat models that are cured by curcumin, Nrf2 activation prevented glomerular hypertension, hyperfiltration, and decreased the antioxidant enzyme [110]. Therefore, nephrotoxicity has been effectively protected via activating Nrf2 by the curcumin therapeutic effect [111,112].

### 7.2. Neurological Diseases

Oxidative stress and neuroinflammation are closely associated with ALS (Amyotrophic lateral sclerosis), AD (Alzheimer’s), PD (Parkinson’s), MS (Multiple sclerosis), and HD (Huntington’s disease) [113,114]. Nrf2 signaling pathway is a potential therapeutic purpose for neuroinflammatory and neurodegenerative disorders. Nrf2, by the role in the induction of antioxidant proteins and suppressing the Nf-*κ*B inflammatory pathway, has a protective effect in neuroinflammation [115]. Some studies have confirmed that the depletion of Nrf2 in astroglial and neuronal cultures derived from an Nrf2 knock-out mice model has sensitized oxidative stress [8]. Additionally, curcumin exhibited memory-enhancing properties. A moderate content of aggregated Aβ_1–42_ proteins parallel with reduced hippocampal caspase-3 content has been observed in curcumin-treated mice. Besides, Nrf2 translocation in nuclear was significantly enhanced [115]. Curcumin, by elevating Nrf2 and down-regulation of NF-*κ*B, could reduce brain edema and neurological dysfunction after cerebral ischemia/reperfusion in the rat [116]. A rotenone-mediated PD model of rats that were pre-treated with curcumin could suppress the oxidative stress via the Akt/Nrf2 signaling pathway activation and the upregulation of HO-1 and QOD-1 protein expression [61]. Traumatic brain injury (TBI) is the main reason for death and disability worldwide, and it consumes many medical resources. Inflammation, calcium homeostasis, oxidative stress, and the disruption of the blood–brain barrier are secondary injuries after TBI. In one study in a mouse model, the neuroprotective effects of curcumin on TBI have been investigated. The administration of curcumin reduced cerebral edema, boosted neurological function, and suppressed neuronal apoptosis in a dose-dependent manner. Curcumin promoted the translocation of Nrf2 from the cytoplasm to the nucleus, activated the Nrf2/ARE signaling pathway, and provided neuroprotection in the TBI model [117]. Arsenic-triggered toxicity in PC12 cells pre-treatment by curcumin 2.5 *μ*M has effectively been cured against cytotoxicity by increasing cell viability via inducing the Nrf2 antioxidant signaling pathway [118]. Another study showed that curcumin pre-treatment could reduce amyloid-β in human IMR-32 neuroblastoma cells by activating the Nrf2 and (Apurinic/apyrimidinic endonuclease 1) APE1 protein-mediated pathways [119].

### 7.3. Liver Diseases

Recent studies have indicated that curcumin could activate nuclear translocation of Nrf2 and upregulate downstream enzymes in order to protect rats from lipopolysaccharide/D-galactosamine-induced acute liver injury. In three days, curcumin administration could attenuate hepatic pathological damage, as well as reduce malondialdehyde (MDA), serum alanine aminotransferase (ALT), and aspartate aminotransferase (AST) content in experimental rats. The molecular-level investigation demonstrated that curcumin could upregulate the expression of nuclear Nrf2 and Nrf2-dependent antioxidant defense genes, including heme oxygenase-1, quinone, NAD(P)H dehydrogenase, and glutamate-cysteine ligase (GCLC); therefore, it could inhibit the activation of NF-*κ*B in a dose-dependent manner [120]. The curcumin treatment of rat hepatic stellate cells (HSCs)-T6 has demonstrated significantly decreased ROS and MDA contents and increased the levels of GSH and nuclear expression levels of Nrf2 [121]. The oxaliplatin-based chemotherapy induces liver injury in patients via the generation of oxidative stress. Curcumin treatment could alleviate hepatic pathological damage and splenomegaly by activating the Nrf2 pathway, inhibiting oxidative stress, inflammation, and coagulation system. It could increase the expression of HO-1 and NOQ1 in mice [122]. Studies have shown that ethanol-induced oxidative damage in hepatocytes could protect by the Nrf2 activation pathway [123]. Ethanol could increase the liver organ coefficient, serum ALT levels, irregular arrangement of hepatocytes, and the disappearance of hepatic cord in the rat model. The combination of curcumin and Baicalin in alcoholic liver disease treatment promoted the downstream antioxidant enzymes NQO1 and HO-1 expression by activating the Nrf2-mediated signaling pathway, and it had a further protective effect on liver toxicity [124]. 

### 7.4. Diabetes 

The significant health concern is diabetes mellitus worldwide. Metabolic syndrome, nephropathy, obesity, retinopathy, and neuropathy are under the role of Nrf2; hence, Nrf2 activation prevents the progression of diabetes and its complications [125]. In one study, glucose intolerance improved in high fat diet-fed mice by curcumin treatment. In these mice, curcumin effectively modified muscular oxidative stress and decreased malondialdehyde by activating Nrf2 [126]. Demethoxy curcuminoids could induce cellular defense mechanisms, like the HO-1 signaling pathway after translocating Nrf2 to the nucleus of β-cells of mouse islet under conditions of stress, as seen in diabetes [127]. The effect of curcumin on high glucose-induced in the kidney of rat (diabetic nephropathy) was investigated. The result showed that curcumin increased the levels of Nrf2 and HO-1, which could promote the anti-fibrotic effects [111]. Antioxidants, like curcumin, increase ghrelin secretion. Ghrelin is a peptide hormone in the stomach with many actions, including the regulation of food intake, adiposity, blood glucose, and body weight. Data showed that Nrf2 was involved in ghrelin secretion. By activating the Nrf2, curcumin could increase ghrelin secretion and block the inhibitory effects of glucose on ghrelin secretion [128]. A complex process, like energy and redox balance, could achieve by modulating a cytoprotective regulator effect of transcription factors, like the Nrf2 protein. This signaling pathway could introduce a new strategy for overcoming obesity [129]. 

### 7.5. Cancer 

Chemoresistance is the main hindrance in the operation of tumors. ROS could induce some of the signals that lead cancer cells to evade apoptosis by alterations in the expression of genes that modulate the signaling of the cell cycle and DNA repair. Nrf2 has tumor-suppressive and tumor-promoting action. The cytoprotective properties of Nrf2 occur in transient activation, but continual activation may cause tumor progression and tumor resistance to therapies. Hence, Nrf2 overexpression in cancer cells is considered to be a marker of chemoresistance [130]. Some studies have indicated that curcumin could sensitize cancer cells through the activation of Nrf2 [78,131]. Platinum-based agents, like cisplatin, are used for various malignancies cancer treatment, and it has some limitations, such as chemoresistance and ototoxic side effects. Curcumin, as an adjuvant to chemotherapeutic, could upregulate the Nrf2/HO-1 pathway and downregulate p53 phosphorylation. Curcumin could influence inflammatory pathways that counteract NF-*κ*B activation. Curcumin has the biphasic activity of antioxidants in normal cells undergoing stressful conditions and pro-oxidant in cancer cells; these polyphenols probably engage an interplay among the key factors Nrf2, NF-*κ*B, STAT3, and p53 [132]. Skin is highly vulnerable to damage by environmental toxicants, like UV, chemical carcinogens, and infectious agents. The results of one study showed that mouse epidermal JB6 cells by curcumin treatment induced the expression of Nrf2 protein and HO-1 in a concentration and time-dependent manner [71]. In the search for studies, it has been revealed that inhibitors of the Nrf2 pathway could reduce the proliferative and survival of cancer cells and could also sensitize tumors to chemo- and radiotherapy. For a drug design strategy based on the Nrf2 pathway in cancer therapy, it must be considered that the overexpression of the Nrf2 pathway has also linked to tumorigenesis. Hence, in different phases of clinical trials must utilize both synthetic and natural Nrf2 agonists or activators [1,33,133]. 

## 8. Anti-Viral Effects of Curcumin

Seeking the repurposing of approved pharmaceuticals or natural compounds could be a reasonable strategy for designing novel anti-viral drugs. Curcumin has been reported to be effective against viral infection through specific and non-specific manners of action (Table 1). It specifically exhibits an anti-viral effect by inhibiting particular viral proteins or suppressing special gene expression. Moreover, several studies have indicated that curcumin could inhibit numerous viral infections via direct interference with the viral replication machinery or suppressing cellular signaling pathways that are essential for viral replication. In some cases, curcumin inhibited the viral haemagglutination (HA) function and interrupted the early stage of infection by abrogating virus-cell attachment. Hemagglutinin (HA) is the main capsid glycoprotein of the influenza virus that mediates virus attachment [134]. Examples of specific anti-viral activities by curcumin include: anti-human immunodeficiency virus (HIV) activity by inhibiting the HIV integrase [135], protease [136] and trans-activator of transcription (Tat) protein [137], anti-(HBV) activity by downregulating peroxisome proliferator-activated receptor-gamma coactivator (PGC-1α) [138], anti-human papillomavirus (HPV) activity by inhibiting E6 and E7 expression [139], anti-herpes simplex virus 1 (HSV-1) activity by reducing the expression of viral immediate-early (IE) genes and RNA polymerase II recruitment to IE gene promoters, anti-human cytomegalovirus (HCMV) activity by downregulating the cellular heat shock protein 90 (HSP90) [140,141,142,143,144,145,146], anti-human T-cell leukemia virus type 1 (HTLV-1) activity by inhibiting the Akt signaling pathway [147] and increasing the expression of c-FLIP [148], and hepatitis C virus (HCV), by the inhibition of the PI3K-Akt signaling pathway and promotion of HO-1 expression [149]. The anti-viral activity mechanism of the curcumin against Coxsackie virus B3 (CVB3), Dengue virus type 2 (DNV-2), enteroviruses 71 (EV71), and Japanese encephalitis virus (JEV) is the inhibition of the viral replication through the dysregulation of ubiquitin-proteasome system (UPS) in host eukaryotic cells [141,150,151,152,153].

Curcumin is a hydrophobic compound and it affects the nonspecifically the fluidity of the viral envelope or alters the fusion ability and rigidity of the host plasma membrane and interferes with the binding and fusion with the host plasma membrane, thus inhibiting the entry of numerous viruses to the host cells, including: hepatitis C virus (HCV) [154], Bovine herpesvirus type 1 (BoHV-1) [160], and human norovirus (HuNoV) [161]. Chikungunya virus (CHIKV) and Zika virus (ZIKV) are enveloped RNA viruses and they belong to the alphavirus and flavivirus families, respectively. Both of them are mosquito-transmitted and they cause severe diseases with symptoms, like fever and arthritis or neuropathologies and microcephaly. Curcumin treatment reduced their infectivity by interfering and blocking the viral entry in a time-and dose-dependent manner without influencing on the cellular viability and viral RNA degradation viral envelope [162,163].

### Curcumin Prevents Viral Infection Via Modulating Nrf2

Most viral infections are accompanied by ROS generation, which activates the Nrf2/HO-1 signaling pathway in the host cells in order to protect infected cells against oxidative stress via anti-inflammation and antioxidant activities. Therefore, it involves the suppression of multiple viral infections. Some examples are, as follows: hepatitis C virus (HCV), enterovirus 71 (EV71), human immunodeficiency virus (HIV), hepatitis B virus (HBV), and ebolavirus (EBOV) [7]. 

Marburg virus (MARV) is Filoviridae that causes severe hemorrhagic fever with a high mortality in humans. The acidic loop of the virus’s VP24 protein interacts with the Keap1 Kelch domain with a high affinity to activate Nrf2, followed by the expression of antioxidant genes to combat viral pathogenesis and replication [155].

In Nrf2 knock-out mice, pro-inflammatory genes that are regulated by NF-*κ*B are highly expressed. For example, in Nrf2-deficient mice, viral clearance attenuated, and NF-*κ*B activity increased in response to the respiratory syncytial virus (RSV) [4]. The highly infectious respiratory virus of Influenza A (IAV) usually causes acute respiratory distress syndrome (ARDS) and acute lung injury with considerable morbidity and mortality, despite vaccine production. Infection by IVA is accompanied by ROS generation which is correlates with the activation of PI3K/Akt and the downstream cascade of toll-like receptor (TLR) signaling pathway, mitogen-activated protein kinase (MAPK or MAP kinase), and nuclear factor-*κ*B (NF-*κ*B), and decreasing the activity of Nrf2/HO-1 pathway. Curcumin could inhibit IAV replication by activating the Nrf2/HO-1 signaling pathway that could inhibit TLR4 expression and downregulate PI3K/Akt, MAPK, and NF-*κ*B pathway. Therefore, curcumin could directly block adsorption and inhibit IVA proliferation [156]. The infected cell treated with curcumin could suppress EV71 replication at the early stage by inhibition of the synthesis of viral RNA and the expression of viral needed host proteins Golgi-specific brefeldin A-resistance guanine nucleotide exchange factor 1 (GBF1) and phosphatidylinositol 4-kinase beta (PI4Kβ) and activate the ERK signaling pathway [153].

The cytokine storm plays a critical role in the progression of fatal pneumonia, including recent pandemics, such as severe acute respiratory syndrome-related coronavirus (SARS-CoV), middle east respiratory syndrome-related coronavirus (MERS-CoV), and SARS-CoV2. The overactivation of immune cells produces a cytokine storm and causes acute lung injury or fatal acute respiratory distress syndrome. Curcumin could inhibit the production of inflammatory cytokines by upregulating anti-inflammatory Interleukin 10 (IL-10), which prevents the NF-*κ*B signaling in macrophages and activating Nrf2/HO-1 signaling in order to reduce oxidative stress, which ameliorates viral pneumonia [158].

Infection by the respiratory syncytial virus (RSV) stimulates oxidative stress through decreasing the cellular level of Nrf2 by Keap1 signaling independent ubiquitination [157]. The viral infection brings about cellular damage and lung inflammation. Curcumin could ameliorate RSV-induced clinical symptoms by inhibition of the RSV replication via the activation of TNFα signaling and NF-*κ*B phosphorylation and activation of protein kinase RNA-activated (PKR) that regulates the activation of the translation initiation factor eIF-2α replication [164].

## 9. Potential Mechanism of Curcumin on Pandemic Virus COVID-19

The over-activation of immune cells and hyper-production of cytokines, which is known as a cytokine storm or cytokine release syndrome (CRS), results in acute lung injury or fatal ARDS. The pandemic disease that is caused by SARS-CoV2 infection is named coronavirus 2019 (COVID-19), which is associated with CRS and characterized by exacerbated pro-inflammatory cytokines release (cytokine cascade). Nrf2 activation plays a role in both the execution of inflammation and decreases the intensity of the cytokine storm. However, COVID-19 patients show a suppressed Nrf2 pathway [165]. Nrf2 is a cytoprotective factor that restores redox homeostasis in the cell, and its activation not only promotes the transcription of several macrophage-specific genes that protect the cell against viral infection [166], but also inhibits the expression of the inflammatory cytokines in macrophages [167]. Nrf2 also inhibits the NF-*κ*B that is activated in SARS-CoV2 infected cell [159,166].

Nrf2 reduces angiotensin-converting enzyme 2 (ACE2) receptor expression in respiratory epithelial cells [168]. Recently it has been reported that the Nrf2 activator PB125^®^ downregulates the mRNA expression of ACE2 and transmembrane serine protease 2 (TMPRSS2) [167], which play a vital role in SARS-CoV2 entry into host cells.

More than all, Nrf2 could activate the heme oxygenase 1 (HO-1) signaling pathway that catalyzes the degradation of heme into biliverdin, Fe^2+^, and CO, which are putative anti-SARS-CoV2 activity [169]. Consequently, the pharmacological compound that could activate the Nrf2 might be an effective medicine to cure pandemic COVID-19 disease [166]. Curcumin is a natural compound without a toxic effect that could be a beneficial candidate for ameliorating the COVID-19 disease. One of the proteases that contribute to SARS-CoV2 infection is the main protease (M^pro^), which is a potential target protein for COVID-19 treatment. M^pro^ is vital for the proteolytic maturation of the SARS-CoV2. *In-silico* modeling showed that demethoxycurcumine and curcumin could bind to M^pro^ via H-bond formation [170]. *In-silico* molecular modeling showed that curcumin could attach to spike glycoprotein-receptor binding domain (RBD) and peptidase domain (PD)-ACE2, which are necessary for viral entrance and infection [171].

Moreover, curcumin may be able to prevent the virus entry to the host cell by altering membrane fluidity due to its hydrophobic properties. Curcumin also inhibits the NF-*κ*B through multiple mechanisms. Curcumin acts as an inhibitor of the activation of nuclear factor kappa-B kinase subunit beta (IKKβ), activator of the inhibitory subunit of NF kappa B alpha (IκBα) and 5’ AMP-activated protein kinase (AMPK) [159]. Consequently, curcumin could prevent infection by SARS-CoV2 via direct interaction with viral molecules or indirect effects on transcriptional or other molecules in the host cell, but more studies are required in order to investigate the precise mechanism of curcumin action in COVID-19 as a potential therapeutic.

## 10. Conclusions

Natural products have been used for various therapeutic targets by people around the world since ancient times. Turmeric is a medicinal herb that is applied for the treatment of various diseases in the traditional medicine of China and Indian and Iran. Curcumin is a polyphenolic compound that is extracted from turmeric and it has numerous pharmacological activities, like anti-cancer, anti-diabetic, anti-viral, anti-inflammatory, and antioxidant activities. Curcumin could modify multiple cell signaling pathways and downregulate cell survival gene expression profile by the effects on transcription factors. Nrf2 is a transcription factor that activates numerous antioxidant and detoxifying enzymes in order to regulate oxidative stress in the cell. Nrf2 has a variety of protective effects on toxic, acute, and chronic diseases and viral infection. Nrf2 activators increased the antioxidant response genes, including HO-1 and NQO-1 expression. Curcumin is a non-toxic compound that could modify Cys151 of Keap1. Curcumin could promote the dissociation of Nrf2 from Keap1 and its subsequent nuclear translocation. Screening for Nrf2 activators would lead to the discovery of new pharmaceuticals for oxidative stress-associated chronic diseases. Curcumin’s ability to affect a wide range of molecular targets in the cell or penetrate the lipid bilayer structure makes it a possible candidate to treat viral diseases or common pathologic mechanisms of many chronic diseases that are characterized by oxidative stress and inflammation. The pharmacophore of the curcumin is a distinct blend of Michael acceptor and the antioxidant properties, which make it a candidate for potential medicinal applications. However, low bioavailability, absorption, permeability, and rapid metabolism still obstruct curcumin’s clinical use. The scientists design new strategies for enhancing curcumin therapeutic applications and improving physicochemical properties. This article explained the latest and detailed curcumin study as an Nrf2 therapeutic activator in chronic and viral infected diseases, such as SARS-Cov2. We hoped that this review article motivates scientists’ attention to curcumin as an efficient therapeutic agent for medicinal purposes, especially in oxidative stress-related diseases.

## Figures and Tables

**Table 1:** A specific and non-specific mode of curcumin potential properties for anti-viral activity.

Activity	Target Virus	Mechanism of Action	References
**A specific mode of action**			
Anti-HIV	Human immunodeficiency virus	Inhibition of HIV integrase Inhibition of HIV protease Inhibition of Trans-Activator of transcription (Tat) protein	[135] [136] [137]
Anti-HBV	Hepatitis B virus	Downregulation of peroxisome proliferator-activated receptor-gamma coactivator (PGC-1α)	[138]
Anti-HPV	Human papilloma virus	Inhibition of E6 and E7 expression	[139]
Anti- HTLV-1	Human T-cell leukemia virus type 1	Inhibition of the Akt signaling pathway promotion the expression of c-FLIP	[147] [148]
Anti-HSV-1	Herpes simplex virus 1	Suppression of the expression of viral immediate-early (IE) genes Recruitment of RNA polymerase II to IE gene promoters	[140] [145]
Anti-HCMV	Human cytomegalovirus	Downregulation of the cellular heat shock protein 90 (HSP90)	[146]
Anti-HCV	Hepatitis C virus	Inhibition of the PI3K-Akt signaling pathway Promotion of HO-1 expression	[154] [149]
Anti-CVB3	Coxsackie virus B3	Inhibition of the viral replication through dysregulation of the ubiquitin-proteasome system (UPS) in host eukaryotic cells	[151] [152] [153] [150]
Anti-DNV2	Dengue virus type 2		
Anti-EV71	Enterovirus 71		
Anti-JEV	Japanese encephalitis Virus		
Anti-EV71	Enterovirus 71	Inhibition of viral RNA replication Activate the ERK signaling pathway	[153]
Anti-MARV	Marburg virus	Activation of the Nrf2/HO-1 signaling pathway	[155]
Anti-IAV	Influenza A virus		[156]
Anti-RSV	Respiratory syncytial virus		[157]
Anti-SARS-CoV	SARS- coronavirus		[158]
Anti-MERS-CoV	MERS- coronavirus		[158]
Anti-SARS-CoV2	SARS- coronavirus 2		[159]
**Non-specific mode of action**			
Anti-HCV	Hepatitis C virus	Perturbation of the fluidity of the viral envelope	[154] [160] [161]
Anti-BoHV-1	Bovine herpesvirus type 1		
Anti-HuNoV	Human norovirus		
Anti-CHIKV	Chikungunya virus	Interfering and blocking the viral entry	[162] [163]
